# Long-term storage of blood RNA collected in RNA stabilizing Tempus tubes in a large biobank – evaluation of RNA quality and stability

**DOI:** 10.1186/1756-0500-7-633

**Published:** 2014-09-12

**Authors:** Nur Duale, W Ian Lipkin, Thomas Briese, Jeanette Aarem, Kjersti S Rønningen, Kaja K Aas, Per Magnus, Kari Harbak, Ezra Susser, Gunnar Brunborg

**Affiliations:** The Norwegian Institute of Public Health, PO Box 4404, Nydalen, NO-0403 Oslo Norway; Oslo University Hospital, Rikshospitalet, Oslo Norway; The Mailman School of Public Health, Columbia University, New York, NY USA; New York State Psychiatric Institute, New York, NY USA

**Keywords:** Tempus tubes, Cord blood, Long-term storage, Quality control, RNA stabilization, Biobank

## Abstract

**Background:**

Establishing methods for secure long term storage of RNA is critical to realizing the promise of biobanks in biomedical research. Here, we describe the results of yearly analyses of the same set of umbilical cord and adult whole blood RNA collected in Tempus Blood RNA tubes and stored at -80°C, over a period of up to six years. We systematically investigated the effects of long-term storage of samples (75 Tempus tubes form three adult donors and 30 Tempus tubes from three cord blood donors) on the RNA quality and transcript stability of six selected genes (*CDKN1A*, *FOS*, *IL1B*, *IL8*, *MYC* and *TP53*). This is the first systematic study of both cord and adult blood samples stored for many years.

**Findings:**

The RNA purity and integrity, expressed as RIN-values, were stable up to six years of storage, and there were no storage-related deleterious effects on RNA purity. There were limited intra- and inter-individual variations in RNA yields; however, no consistent trend of decreasing RNA yield was observed with the duration of storage. Some long-term storage effects were found on the relative transcript levels of the six genes when compared to the year 0 samples. However, these changes were within ± 2–fold for both types of blood samples, except for two genes. Our results show that storage of these samples for up to six years did not have significant effects on the RNA quality and transcript stability of the six genes.

**Conclusions:**

Blood RNA is stable in Tempus tubes stored at -80°C over a period of six years. Intact and good-quality RNA suitable for transcript profiling analyses in epidemiological studies was obtained from blood samples stored in Tempus tubes. This suggests that blood samples collected in large biobanks–such as the Mother and Child (MoBa) Cohort at Norwegian Institute of Public Health (NIPH) and frozen in suitable collection tubes for total RNA stabilization, can be used for quantitative studies after at least six years of storage.

**Electronic supplementary material:**

The online version of this article (doi:10.1186/1756-0500-7-633) contains supplementary material, which is available to authorized users.

## Findings

### Background

The Mother and Child (MoBa) is a cohort consisting of more than 114,000 pregnancies recruited between 1999 and 2008 [1]. In addition to detailed questionnaire data, biological materials from the mother, the father and the child (umbilical cord blood) in the form of whole blood and plasma have been collected and are stored together with extracted DNA in a biobank at the Norwegian Institute of Public Health (NIPH) for future use [2].

In 2005, the collection of umbilical cord blood RNA was started as part of the Autism Birth Cohort (ABC) study with support from the National Institute of Neurological Disorders and Stroke (NINDS). The ABC was established to address the natural history of autism spectrum disorders (ASD), explore genetic and pre- or perinatal environmental factors in causation, as well as the interplay between genes and environment, and to facilitate discovery of biomarkers with the potential to enable early recognition and treatment. The ASD cases in the ABC study are identified from the MoBa participants [3]. In MoBa, the cord blood was collected in Tempus Blood RNA tubes [4], which contain stabilizing reagents that preserve RNA transcripts [5]. Samples from hospitals and maternity units all over Norway were transported by standard mail (or by car for shorter distances) to the MoBa Biobank at NIPH in Oslo for storage [4]. There are several reports on *ex vivo* instability of RNA transcripts that may take place during sample collection, transport, storage and RNA extraction [6–8]. Although high-quality RNA can be prepared using fresh blood that is processed immediately, this option is hardly realistic when sampling for a large-scale biobank where the blood samples are collected in remote locations. Therefore, it is critical to reduce the impact of pre-analytical sample handling on RNA quality and stability by stabilizing blood RNA prior to shipping and RNA extraction. This is particularly important when collecting blood samples for large-scale biobanks, where the associated costs are very high. The two commercially available RNA stabilizing technologies PAXgene™ Blood RNA system (PreAnalytiX, QIAGEN/BD, Hombrechtikon, Switzerland), and Tempus™ Blood RNA system (Applied Biosystems, Foster City, CA), where blood is drawn directly into a tube containing RNA stabilizing reagents, are attractive approaches for expression profiling of whole blood due to the simple handling required at the blood collection site.

In MoBa, the Tempus Blood RNA system was selected based on our recent study [4], and approximately 50,000 samples have been collected and stored at –80°C. It is likely that these samples will be kept under these conditions for several more years, whereas selected samples will be processed for measuring gene transcript levels in nested case-control studies. When the collection of Tempus tubes in MoBa started, there were few data available on the effects of long-term storage on RNA quality. Knowing the *ex vivo* instability of RNA transcripts [6–8], it is essential to evaluate the quality of RNA obtained from the stored samples, in the context of potential use in downstream transcriptional profiling studies.

Since this quality control (QC) study would run over several years, probably involving different personnel and different batches of kits, it was important to establish a flexible protocol that was easy to follow but detailed enough to correctly assess factors of importance for gene expression analyses. Here we present the design and results of a QC study to evaluate the effects of long-term storage on blood RNA quality.

### Results and discussion

The NIPH MoBa Biobank was established to collect and curate a wide range of biological samples for future use. RNA is amongst the most labile of biological molecules. We designed and performed a QC study with adult and cord blood RNA to assess the quality of the collected samples over a six year period. Other studies have reported the quality and gene transcript stability of whole blood RNA collected from Tempus tubes [4, 5, 9, 10], but the present report is the first systematic evaluation of long-term storage effects in such samples including both cord and adult blood.

To determine the effects of long-term storage on RNA quality and yield, Tempus Blood RNA samples were taken out of the –80°C storage and total RNA was isolated from each sample, at 0, 1, 2, 4 and 6 years. For each time point (year), 15 Tempus tubes containing adult blood from the same three donors (5 × 3) and six Tempus tubes containing cord blood from three donors (2 × 3) were analyzed for total RNA yield by spectroscopic quantitation, RNA integrity and purity by RIN and OD measurement, and specific transcript stability by real-time qPCR analysis for six cellular genes.

**Figure 1:**
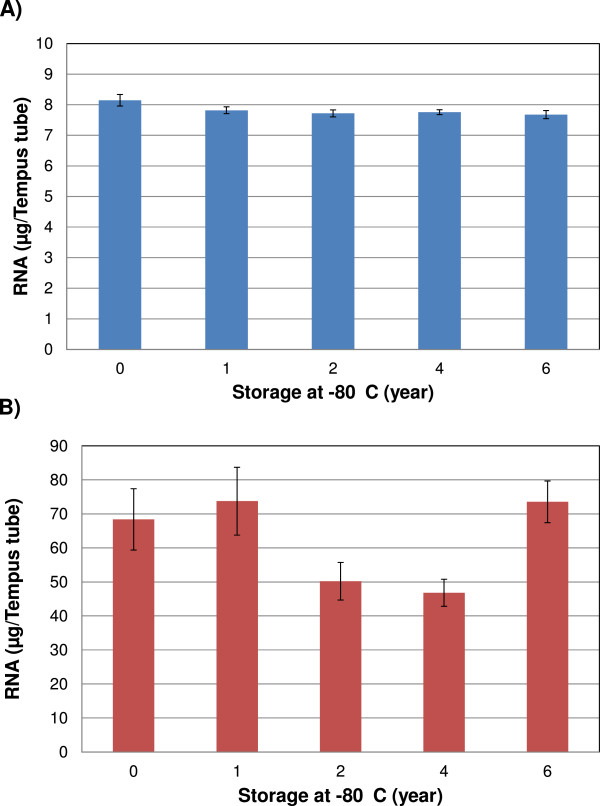
**The effect of long-term storage on RNA yield.** Adult (n = 75 Tempus tubes from 3 donors) and cord (n = 30 Tempus tubes from 3 donors) blood samples were collected in Tempus tubes (3 ml blood per tube) and stored at -80°C for up to six years, followed by total RNA extraction. Each Tempus tube was considered as an independent biological sample. **A)** The RNA yield from the adult blood samples (n = 15 Tempus tubes/year). **B)** The RNA yield from cord blood samples (n = 6 Tempus tubes/year). Significantly lower RNA yields were recorded for cord blood samples analyzed in year 2 and 4. The average total RNA yields for adult and cord blood samples were 7.3 ± 2.6 μg per Tempus tube and 65.5 ± 20.4 μg per Tempus tube, respectively. The RNA yields in cord blood samples were significantly higher than in the adult blood samples. Each bar represents the average RNA yield and the error bar indicates ± SE.

The total RNA yield, as analyzed by NanoDrop™ Spectrophotometer, was 7.3 ± 2.6 μg and 65.5 ± 20.4 μg per Tempus tube, for the adult and cord blood samples, respectively (Figure 1A and B). Consistent with our recent report on effects of sample collection method and short term storage of RNA samples [4], the yields in cord blood samples were significantly higher than in the adult blood samples (data not shown). This difference is likely to reflect the higher cell content of the cord blood [11]. Limited intra- and inter-individual (donor) variations in total RNA yields were observed, but the amounts of RNA were sufficient for downstream gene expression analysis in all adult and cord samples. Significantly lower RNA yields than in years 0, 1 and 6 were recorded for cord blood samples analyzed at year 2 and 4 (Figure 1B). The reason for this is unknown but the lower yields did not seem to be related to the storage time. This may also be related to the different batches of RNA extraction kits. The Tempus tubes were from the same lot number, and all cord blood samples were collected from the three donors on the dates May 3rd, May 18th and May 19th (2005).

**Figure 2:**
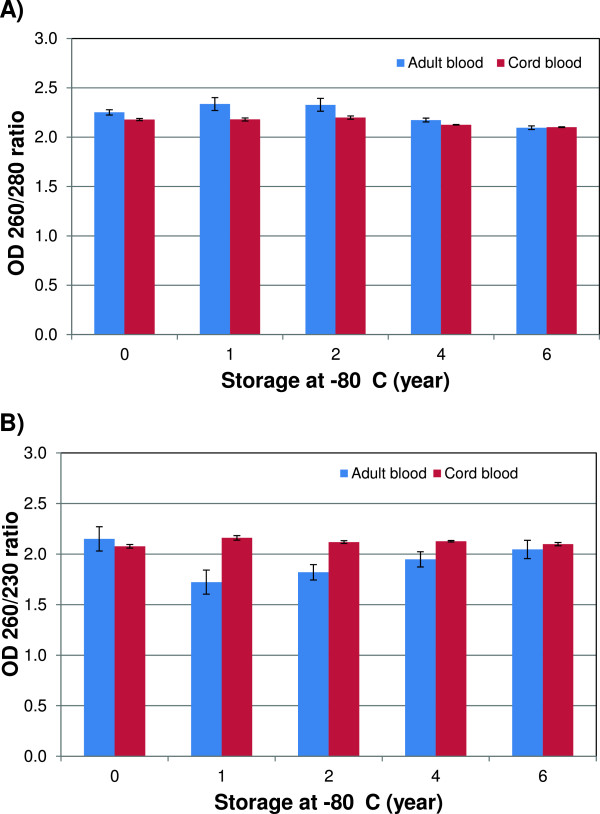
**The effect of long-term storage on RNA purity.** RNA purity of samples isolated from adult (n = 15 Tempus tubes/year) and cord (n = 6 Tempus tubes/year) blood samples collected in Tempus tubes and stored at –80°C until analysis using Nano Drop™. **A)** The OD 260/280 ratios for adult and cord blood samples. **B)** The OD 260/230 ratios for adult and cord blood samples. Each bar represents the average OD-ratios and the error bar indicates ± SE.

The integrity and purity of RNA can be used to evaluate the effects of long-term storage on blood RNA. RNA with an OD 260/280 ratio > 1.8 are generally accepted as pure RNA suitable for gene expression analyses [12], and OD 260/230 ratio < 1.8 generally indicates the presence of contaminants. The OD 260/280 ratios for the samples were within an acceptable range showing greater variation in the adult than in the cord blood samples (Figure 2); however, there were no noticeable storage related deleterious effects on RNA purity.

**Figure 3:**
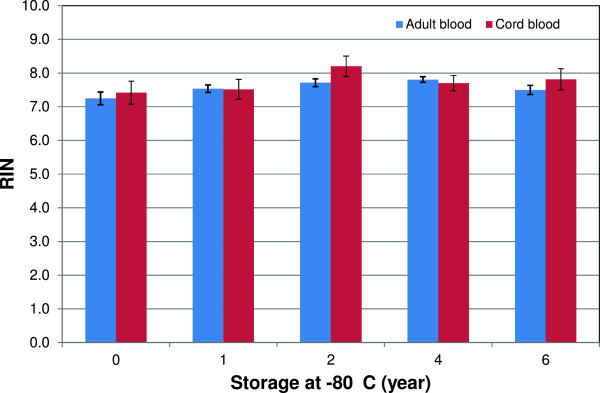
**Long-term storage effects on RNA integrity.** RIN values for RNA samples isolated from Tempus tubes stored at –80°C until analyzed by Agilent 2100 Bioanalyzer. RIN values for adult blood samples (n = 15 Tempus tubes/year) and RIN values for cord blood samples (n = 6 Tempus tubes/year). Bars represent means ± SE. The average RIN values for adult and cord blood samples were 7.6 ± 0.5 and 7.7 ± 0.7, respectively, and no significant long-term storage related effects on RNA integrity were observed.

The effects of long-term storage on RNA integrity, expressed as RIN-values, are shown in Figure 3. The average RIN values for adult and cord blood samples were 7.6 ± 0.5 and 7.7 ± 0.7, respectively. RIN values did not decrease with storage time (Figure 3). RIN values may range from 1 to 10 (RIN = 1; low RNA quality to RIN = 10; highest RNA quality) [13]; RIN-values higher than five imply an acceptable RNA quality, whereas RIN-values above eight are considered ideal for downstream applications [7, 8]. In this QC study, good-quality RNA with average RIN values above seven was obtained from both adult and cord blood samples. In our recent study, we also obtained good-quality RNA from blood samples collected in Tempus tubes [4]. Our results indicate that the quality of RNA obtained from blood samples were stable during storage, although some variability with RIN values was recorded among donors as well as replicate samples from the same donor.

**Figure 4:**
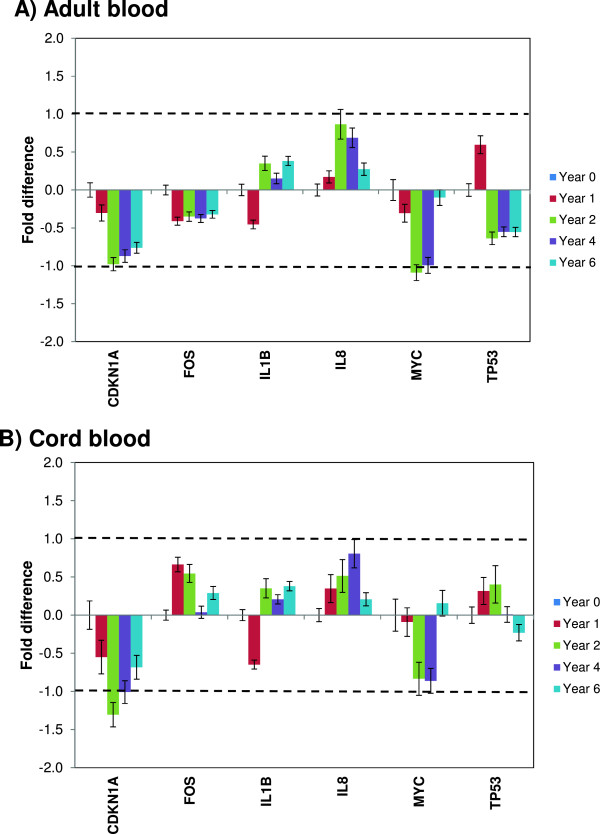
**Long-term storage effects on RNA transcript levels.** The transcript levels for six genes were analyzed by RT-qPCR, using RNA extracted from adult blood and cord blood samples collected in Tempus tubes stored at –80°C until analysis. A total of 21 Tempus Blood RNA tubes (15 adult blood samples and 6 cord blood samples) were analyzed in each year. **A)** Relative transcript levels of six genes from adult blood samples (n = 15 Tempus tubes/year). **B)** Relative transcript levels of six genes from cord blood samples (n = 6 Tempus tubes/year). The year 0 samples were used as reference (calibrators) and all other samples were compared against the reference samples. The dashed lines indicate ± 2-fold. Each bar represents the average log2-transformed fold change values and the error bar indicates ± SE.

The RNA transcript stability in whole blood is reported to be affected by *ex vivo* conditions such as the collection procedure, shipment conditions, storage temperature and duration [4, 6, 14]. In this study, the RNA transcript levels for six selected genes (*CDKN1A*, *FOS*, *IL1B*, *IL8*, *MYC* and *TP53*) were investigated by RT-qPCR and target genes were normalized by 18S rRNA (internal control) (Figure 4A and B). These genes were selected based on our recent study [4] and literature search [15, 16]. Storage of both adult and cord blood samples at -80°C for up to six years was associated with limited time- and sample-dependent changes in RNA transcript stability. For both adult and cord blood samples, an effect of storage was seen for the six genes when compared to the year 0 samples and the effect was more pronounced for *CDKN1A*, *IL8* and *MYC* genes (Figure 4). Furthermore, the *IL1B* and *TP53* transcript levels were different than the other transcripts in year 1, but not in subsequent years. The reasons for the observed variable effects of storage on RNA transcript stability are unclear. These genes, *CDKN1A*, *IL8* and *MYC*, had high average *Cq*-*values* above 30, particularly for the cord blood samples, and the corresponding low expression levels are expected to be associated with larger variability. Gene specific transcript stability may also be affected by secondary structure, and possibly by the amplicon length and the distance of the amplified region in regard to the polyadenylation site. The amplicons lengths for the six genes are all similarly short (66–107 bp) and the locations of the amplified regions were close to 5′-terminus, except for *IL1B*. Nevertheless, the observed transcript level changes were within ± 2–fold for both types of blood samples, except for *CDKN1A* and *MYC* gene at the 2-year storage time point (Figure 4).

**Figure 5:**
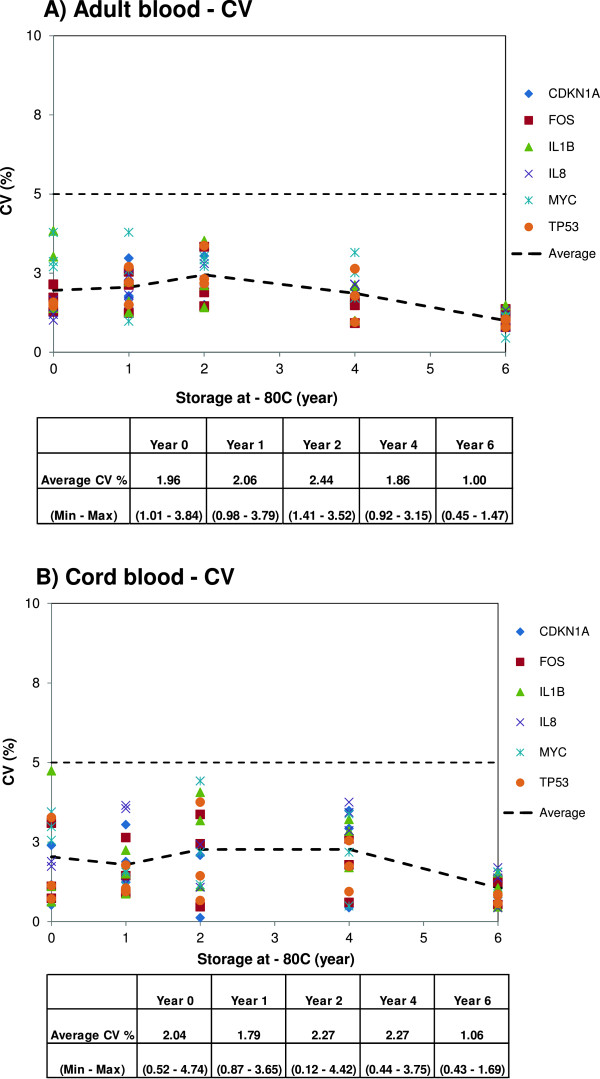
**Variations in average Cq-values within and between donors.** The coefficients of variation (CV) of the average Cq-values were calculated for adult and cord blood samples collected in Tempus tubes and stored at –80°C until analysis for the expression of six genes. The CVs within a donor (intra-donor) and between donors (inter-donor) across the storage years are presented. **A)** Adult blood samples. CVs ranged from 0.45% to 3.84%. **B)** Cord blood samples. CVs ranged from 0.12% to 4.74%. CV of 5% is indicated by a stippled line; CVs for all samples were considerably lower reflecting minor variation in the average Cq-values. Each point represents the average CV of samples from one donor.

We also investigated the variations in the raw non-normalized *Cq*-values by calculating the coefficient of variation (CV) of each gene after each storage year, i.e., mean *Cq* variations were established within a donor (intra-donor) and between donors (inter-donor). Variations were also calculated across all storage years. The non-normalized raw *Cq* and CV values for the six genes are presented in Additional file 1 and Figure 5. From this analysis, an overall picture of long-term storage effects on transcript stability can be obtained. The differences in the raw non-normalized *Cq-values* between stored and year 0 samples for each gene were small indicating low variability (Additional file 1). For adult and cord blood samples, the variations in the average *Cq-values* and CVs within a donor and between donors were small; the CVs ranged from 0.45% to 3.84% and from 0.12% to 4.74%, respectively (Figure 5 and Additional file 1). Furthermore, the variability (CV %) between donors (inter-donor) and between storage years was not higher than the variability within a donor (intra-donor), reflecting the minor variation in expression of these genes (Figure 5). An interesting finding was that samples from storage year 6 had a narrower CV range than samples from the other years (Figure 5); the reason for this is unclear. It has been reported that mean CV values lower than 25% are typically observed for stably expressed reference genes in relatively homogeneous sample panels [17]. The calculated CV values for stored samples were less than 5.0% for all samples indicating very low variability (Figure 5). The feasibility of detecting low abundant RNA transcripts in blood samples collected in the Tempus tube was evaluated. Low abundant transcripts, i.e., transcripts with *Cq-*values above 30 cycles, (*CDKN1A*, *IL8* and *MYC*) were detected in all analyzed samples (Additional file 1). Recently, mRNA targets have been detected with varying abundance levels from blood sample collected in Tempus tubes without globin mRNA reduction using qPCR assays [18]. The interference of the high percentage of globin transcripts from red blood cells (RBC) – which constitute ~70% of the whole blood mRNA – may decrease the sensitivity of detecting less abundant mRNA transcripts, particularly in the microarray and Next-generation sequencing technologies [19, 20]. The RBC globin mRNA transcripts in the RNA population can be removed with an enzymatic depletion using commercially available globin RNA reduction kits, but this reaction reduced the quality of total RNA and is not always recommended [21]. Taken together, our results demonstrate that RNA transcripts which are otherwise highly sensitive to *ex vivo* conditions are efficiently protected and remain stable during long-term storage in Tempus tubes.

### Conclusions

Blood cell RNA appears to be stable in Tempus tubes stored for up to six years at –80°C. Intact and good-quality RNA suitable for transcript profiling analyses in epidemiological studies can be obtained from blood samples stored in Tempus tubes. This suggests that blood samples in large biobanks – such as the MoBa Biobank at NIPH – and frozen in suitable collection tubes for total RNA stabilization, can be used for quantitative studies after several years of storage.

### Methods

#### Sample collection and experimental design

Whole blood samples for RNA quality assessment were collected from three healthy, consenting adult volunteers among the NIPH staff and from the umbilical cord of three newborns whose mothers had given their informed consent to participate in MoBa. The samples were collected into Tempus tubes (3 ml blood per tube) according to the manufacturer’s instructions (Applied Biosystems, Foster City, CA). For long-term storage QC, 170 Tempus tubes were collected from three (A1, A2 and A3) adult blood donors and 82 Tempus tubes from three (C4, C5 and C6) cord blood donors. The tubes were randomized for each donor and labeled with unique numbers. The adult blood samples were kept at ambient temperature for two hours before they were frozen at –20°C overnight and then transferred to –80°C for long-term storage. The cord blood samples were shipped from a maternity unit at Ullevål University Hospital at ambient temperature to NIPH within one day but were otherwise handled in the same way as samples from the adults. For each time point (year), 15 Tempus tubes containing adult blood from the three donors (5 × 3) and six Tempus tubes containing cord blood from the three donors (2 × 3) were analyzed; each Tempus tube was considered as an independent biological sample. The rational for this experimental design was to establish a flexible protocol that was easy to follow but detailed enough to correctly assess long-term storage effects on RNA quality. This QC study would run over several years, probably involving different personnel and different batches of kits. Therefore, it was important to collect enough blood samples from each donor and analyze several replicates from each donor at each time point in order to control for donor-to-donor variations and variations that may take place between phlebotomy and storage. Of the 170 adult and 82 cord blood samples which were collected in 2005, 75 Tempus tubes from three adult donors and 30 tubes from three cord blood donors have so far been analyzed. Since each Tempus tube was considered as an independent biological sample, the analysis of in total 105 Tempus tubes should provide a good estimate on the quality and stability of the samples stored in the Biobank. The Regional Ethics Committee and the Data Inspectorate approved the MoBa study, and participants gave their informed consent.

#### RNA extraction and QC

The samples were thawed overnight at 4°C before RNA extraction the following day. Total RNA was extracted using the Tempus 6-port RNA Isolation Kit Protocol with kit reagents and consumables in a 6100 Nucleic Acid Prep Station according to manufacturer’s instructions, including DNase I treatment and addition of 1× PBS adjusting the total volume to 12 ml prior to processing (Applied Biosystems, Foster City, CA). An aliquot of 5 μl of each extracted total RNA was used for RNA quality control assessments, while the remaining RNA sample was stored at –80°C for later use.

The concentration of extracted total RNA was measured using NanoDrop ND-1000 spectrophotometer (Fisher Scientific, Oslo, Norway). RNA purity was estimated from the OD 260/280 and the OD 260/230 ratios. The RNA integrity was assessed by an Agilent 2100 Bioanalyzer using the Eukaryote total RNA 6000 Nano LabChip kit and Eukaryote total RNA Nano assay according to the manufacturer’s instructions (Agilent Technologies, Palo Alto, CA).

#### Quantitative Real-time PCR (RT-qPCR)

The cDNA was synthesized from 500 ng of total RNA using the High Capacity cDNA Reverse Transcription Kit, without RNase Inhibitor (Applied Biosystems). Reactions were incubated in an Eppendorf MasterCycler (Eppendorf, Hamburg, Germany) using the following incubation profile: 10 minutes at 25°C, 2 hours at 37°C and finally, 5 minutes at 85°C. The cDNA quality was assessed using the NanoDrop ND-1000, before storing the cDNA at –20°C.

Quantitative real-time PCR was performed in 96 well PCR plates on a Fast 7500 Real Time PCR system (Applied Biosystems). Serial dilutions of cDNA were prepared to determine an appropriate cDNA dilution. A 1:10 dilution of cDNA was mixed with primers and TaqMan^®^ Fast Universal PCR Master Mix, No AmpErase UNG, according to the manufacturer’s protocol (Applied Biosystems). cDNA from each sample was run in triplicate for each gene of interest. No template controls (NTC) were included for each target gene in each run. Transcript levels for the following six genes were measured, applying commercial primers and probe assays from Applied Biosystems: *CDKN1A* (PN: Hs00355782_m1; amplicon size, 66 bp; assay location, 566; exon boundary, 2–3 exon), *FOS* (PN: Hs00170630_m1; amplicon size, 77 bp; assay location, 352; exon boundary, 1–2 exon), *IL1B* (PN: Hs00174097_m1; amplicon size, 94 bp; assay location, 554; exon boundary, 5–6 exon), *IL8* (PN: Hs00174103_m1; amplicon size, 101 bp; assay location, 222; exon boundary, 1–2 exon), *MYC* (PN: Hs00153408_m1; amplicon size, 107 bp; assay location, 1325; exon boundary, 2–3 exon) and *TP53* (PN: Hs00153340_m1; amplicon size, 81 bp; assay location, 173; exon boundary, 1–2 exon). *18S rRNA* (PN: Hs99999901_s1; amplicon size, 187 bp; assay location, 604; exon boundary, 1–1 exon) was used as internal control. These genes were selected based on our recent study [4] and literature search [15, 16] with varying mRNA transcript abundance (from low to high abundant targets). Pooled cDNA was included in each 96-well plate to allow for control of run-to-run variations (data not shown). The 7500 Fast PCR cycling program included an enzyme activation step at 95°C for 20 seconds, and then 40 cycles of annealing and extension steps at 95°C for 3 seconds and 60°C for 30 seconds, respectively.

#### Data analysis

The quantification cycle (*Cq*) values were recorded with SDS v1.3 software (Applied Biosystems, Foster City, CA); the *Cq* value is the fractional cycle number at which the fluorescence exceeds a fixed threshold. The raw *Cq*-values were then exported into Excel-files and analyzed by the comparative *Cq*-method [22, 23] using 18S rRNA as internal control. Prior to normalization, the raw data (*Cq*-values) generated from qPCR experiments were pre-processed to ensure that measurements at low levels were well within the linear area of detection; *Cq-values* radically different from other technical replicates were classified as outliers and excluded. The excluded outliers were replaced by using the information contained in the replicates when available. In addition, all *Cq*-values above 35 were considered beyond the limit of detection (LOD) and coded as missing values, because *Cq-values* above 35 cycles are in general not reliable. Target genes were normalized to 18S rRNA internal controls, [this is given by Δ*Cq*; where ∆*Cq* (sample) = *Cq* (target gene) – *Cq* (internal controls)]. The ΔΔ*Cq* values were generated by subtracting the Δ*Cq*-value for the reference samples (calibrators; year 0 samples) from the Δ*Cq*-value for the samples [∆∆*Cq* = ∆*Cq* (sample) – ∆*Cq* (calibrator); fold change = 2^-∆∆*Cq*^]. The fold change values were then log2-transformed in order to make the values symmetrical around zero. The 18S rRNA stability was evaluated and results are presented in Additional file 2.

#### Statistical analysis

Statistical analysis of RNA yield, purity, integrity and ∆*Cq*-values was carried out by one-way analysis of variance (ANOVA), followed by *post hoc* Dunnett’s tests to allow for multiple comparisons or by non-parametric Kruskal-Wallis test. Normal distribution and equality of variances were evaluated using the Shapiro-Wilk test and the Levene’s test of homogeneity of variance. The data are presented as means ± SE. Statistical analyses were performed using SPSS v17 software (SPSS, Inc., Chicago, IL), and results with p < 0.05 were accepted as statistically significant. The coefficient of variation (CV) of the non-normalized raw *Cq-*value for each gene was calculated by dividing the mean *Cq*-value with the standard deviation. CV, expressed as a percentage, was used for computing the degree of variation in the mean *Cq-values* of the stored samples.

## Electronic supplementary material

Additional file 1: **The non-normalized raw** ***Cq-values.*** The non-normalized raw *Cq-values* for adult (three donors: A1, A2 and A3) and cord blood (three donors: C4, C5 and C6) samples collected in the Tempus tubes and stored for up to six years at -80°C; A) CDKN1A – average *Cq* for adult blood samples was 31.20 ± 1.08 (28.61–33.23), while for cord blood samples was 32.39 ± 1.17 (30.18–34.35); B) FOS – average *Cq* for adult blood samples was 28.20 ± 0.89 (25.38–29.84), while for cord blood samples was 28.75 ± 0.77 (26.72–30.31); C) IL1B – average *Cq* for adult blood samples was 28.96 ± 0.92 (25.77–30.80), while for cord blood samples was 29.68 ± 0.90 (26.38–31.79); D) IL8 – average for adult blood samples *Cq* was 31.29 ± 0.67 (29.39–32.97), while for cord blood samples was 32.46 ± 0.99 (30.18–34.66); E) MYC – average *Cq* for adult blood samples was 29.53 ± 1.23 (26.14–31.81), while for cord blood samples was 30.62 ± 1.23 (27.78–33.41); F) TP53 – average *Cq* for adult blood samples was 27.93 ± 1.05 (25.30–29.93), while for cord blood samples was 29.06 ± 0.72 (27.45–30.91). Each bar represents the average *Cq-values* and the error bar indicates ± SD. G) Transcript abundance of the six genes. (PDF 73 KB)

Additional file 2: **The evaluation of 18S rRNA stability.** The stability of 18S rRNA for adult and cord blood samples were evaluated, and the raw *Cq-values* for 18S rRNA were relatively stable following the storage of the tubes for up to six years at -80°C. A) Adult blood samples (three donors: A1, A2 and A3); average *Cq* was 14.63 ± 0.51 and varied (13.9–16.3) and B) cord blood samples (three donors: C4, C5 and C6); average *Cq* was 14.92 ± 0.86 and varied (13.9–16.9). Each bar represents the average *Cq*-values and the error bar indicates ± SD. (PDF 33 KB)

## Abbreviations

Cq: Quantification cycle threshold
MoBa: the Norwegian Mother and Child Cohort Study
NIPH: Norwegian Institute of Public Health
QC: Quality control
RT-qPCR: Quantitative real-time PCR
RIN: RNA integrity number.
