# Supplementary material for: Long-term storage of blood RNA collected in RNA stabilizing Tempus tubes in a large biobank – evaluation of RNA quality and stability
Source: BMC Res Notes. 2014 Sep 12;7:633. doi: 10.1186/1756-0500-7-633 (PMC4168124; doi:10.1186/1756-0500-7-633)
Supplement: Supplementary file 1 — Additional file 1: The non-normalized raw Cq-values. The non-normalized raw Cq-values for adult (three donors: A1, A2 and A3) and cord blood (three donors: C4, C5 and C6) samples collected in the Tempus tubes and stored for up to six years at -80°C; A) CDKN1A – average Cq for adult blood samples was 31.20 ± 1.08 (28.61–33.23), while for cord blood samples was 32.39 ± 1.17 (30.18–34.35); B) FOS – average Cq for adult blood samples was 28.20 ± 0.89 (25.38–29.84), while for cord blood samples was 28.75 ± 0.77 (26.72–30.31); C) IL1B – average Cq for adult blood samples was 28.96 ± 0.92 (25.77–30.80), while for cord blood samples was 29.68 ± 0.90 (26.38–31.79); D) IL8 – average for adult blood samples Cq was 31.29 ± 0.67 (29.39–32.97), while for cord blood samples was 32.46 ± 0.99 (30.18–34.66); E) MYC – average Cq for adult blood samples was 29.53 ± 1.23 (26.14–31.81), while for cord blood samples was 30.62 ± 1.23 (27.78–33.41); F) TP53 – average Cq for adult blood samples was 27.93 ± 1.05 (25.30–29.93), while for cord blood samples was 29.06 ± 0.72 (27.45–30.91). Each bar represents the average Cq-values and the error bar indicates ± SD. G) Transcript abundance of the six genes. (PDF 73 KB) [file 13104_2014_3158_MOESM1_ESM.pdf]

Additional file 1 - The non-normalized raw *Cq* – values

The non-normalized raw *Cq*–values for adult (three donors: A1, A2 and A3) and cord blood (three donors: C4, C5 and C6) samples collected in Tempus tubes and stored for up to six years at -80ºC. A) CDKN1A – average and range of Cq 31.20 ± 1.08 (28.61 – 33.23), adult blood samples; 32.39 ± 1.17 (30.18 – 34.35), cord blood samples. B) FOS – average and range of Cq 28.20 ± 0.89 (25.38 – 29.84), adult blood samples; 28.75 ± 0.77 (26.72 – 30.31), cord blood samples. C) IL1B – average and range of Cq 28.96 ± 0.92 (25.77 – 30.80), adult blood samples; 29.68 ± 0.90 (26.38 – 31.79), cord blood samples. D) IL8 – average and range of Cq 31.29 ± 0.67 (29.39 – 32.97), adult samples; 32.46 ± 0.99 (30.18 – 34.66), cord blood samples. E) MYC – average and range of Cq 29.53 ± 1.23 (26.14 – 31.81), adult blood samples; 30.62 ± 1.23 (27.78 – 33.41), cord blood samples. F) TP53 – average and range of Cq 27.93 ± 1.05 (25.30 – 29.93), adult blood samples; 29.06 ± 0.72 (27.45 – 30.91), cord blood samples. Average *Cq*-values and error bars (±SD) are shown. G) Transcript abundance of the six genes.

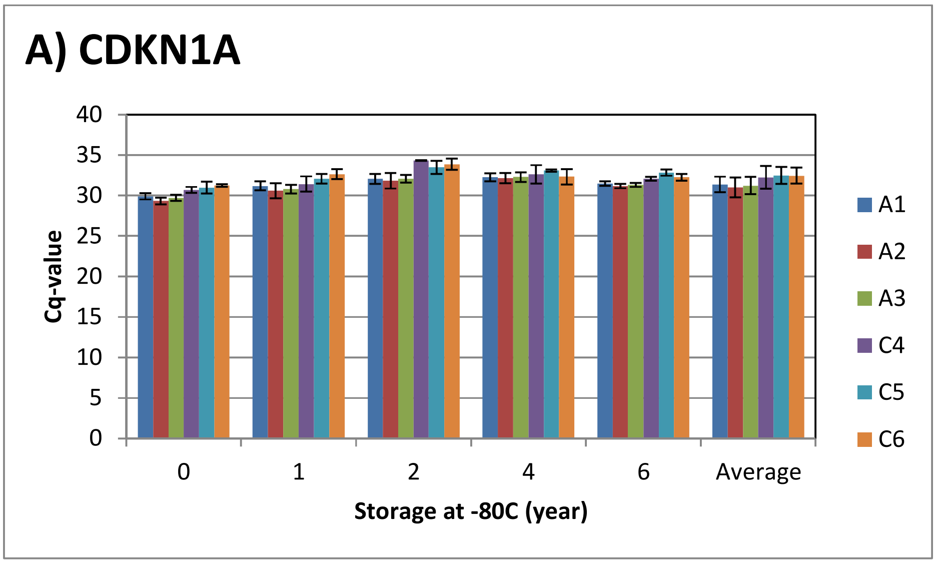

B) FOS

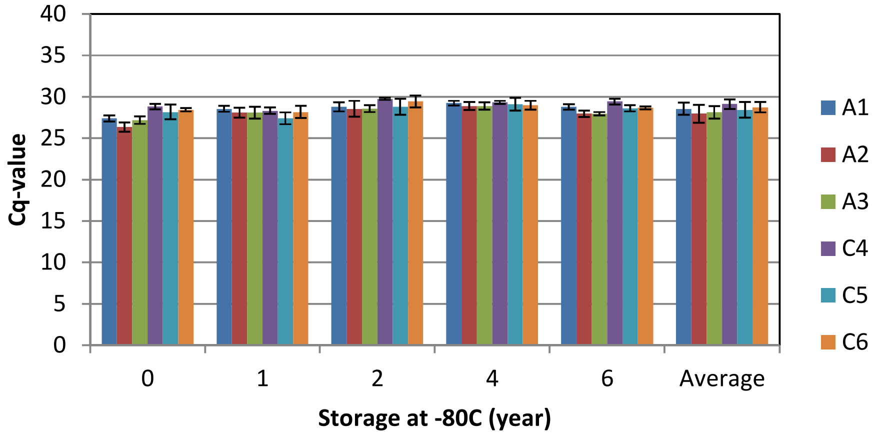

40
35
30
25
20
15
10
5
0
Cq-value
0
1
2
4
6
Average
Storage at -80C (year)
A1
A2
A3
C4
C5
C6

C) IL1B

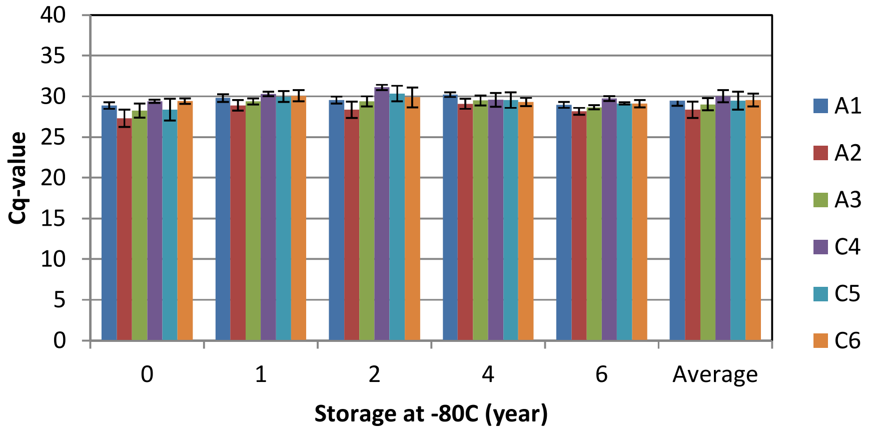

40
35
30
25
20
15
10
5
0
Cq-value
0
1
2
4
6
Average
Storage at -80C (year)
A1
A2
A3
C4
C5
C6

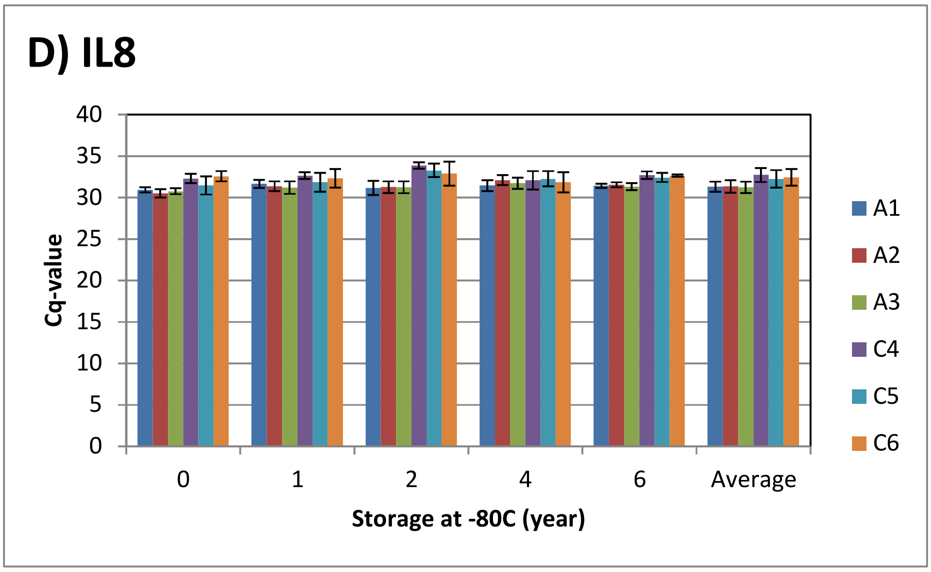
D) IL8
40
35
30
25
20
15
10
5
0
Cq-value
0
1
2
4
6
Average
Storage at -80C (year)
A1
A2
A3
C4
C5
C6

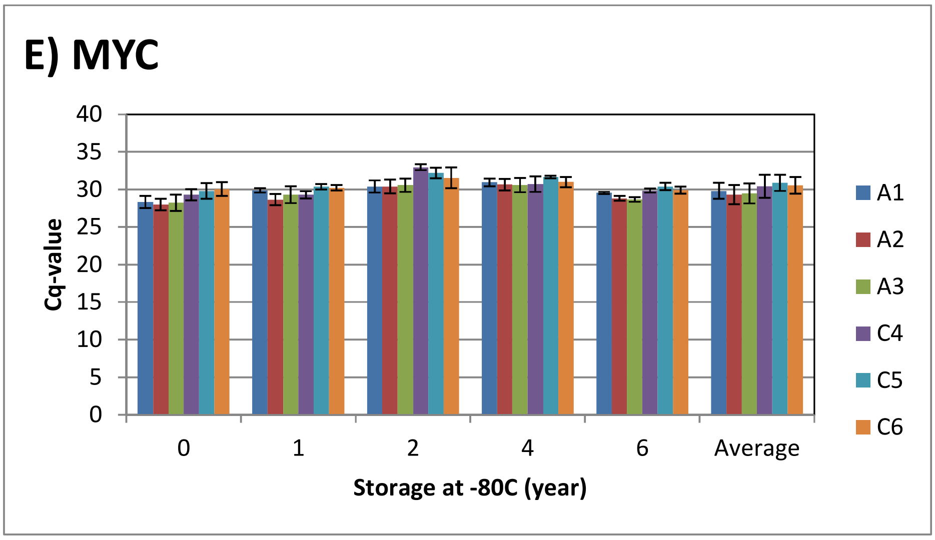
E) MYC
40
35
30
25
20
15
10
5
0
Cq-value
0
1
2
4
6
Average
Storage at -80C (year)
A1
A2
A3
C4
C5
C6

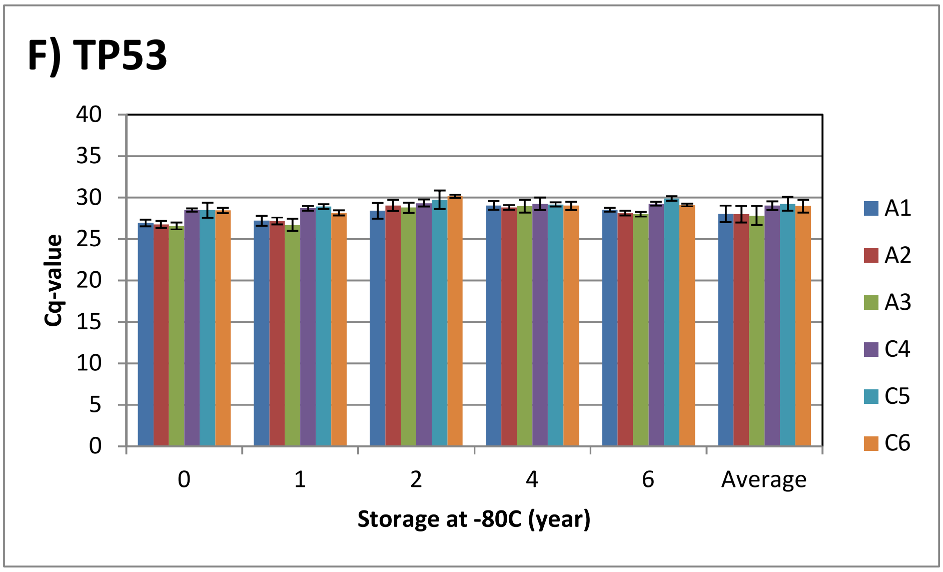

G) Transcript abundance of the six genes.

Gene Symbol	Adult blood	Cord blood	Target Abundance
	Average Cq ± SD	Average Cq ± SD	
18S rRNA (RN18S1)	14.73 ± 0.73	14.95 ± 0.95	High (Cq below 25)
CDKN1A	31.39 ±1.29	32.63 ± 1.50	Low (Cq above 30)
FOS	28.38 ± 1.02	29.17 ± 1.49	Medium (Cq between 25 and 30)
IL1B	29.27 ± 1.22	30.08 ± 1.54	Medium (Cq between 25 and 30)
IL8	31.50 ± 0.92	32.76 ± 1.41	Low (Cq above 30)
MYC	29.79 ± 1.55	30.99 ± 1.72	Low (Cq above 30)
TP53	28.01 ± 1.14	29.50 ± 1.57	Medium (Cq between 25 and 30)
