# Supplementary material for: Long-term storage of blood RNA collected in RNA stabilizing Tempus tubes in a large biobank – evaluation of RNA quality and stability
Source: BMC Res Notes. 2014 Sep 12;7:633. doi: 10.1186/1756-0500-7-633 (PMC4168124; doi:10.1186/1756-0500-7-633)
Supplement: Supplementary file 2 — Additional file 2: The evaluation of 18S rRNA stability. The stability of 18S rRNA for adult and cord blood samples were evaluated, and the raw Cq-values for 18S rRNA were relatively stable following the storage of the tubes for up to six years at -80°C. A) Adult blood samples (three donors: A1, A2 and A3); average Cq was 14.63 ± 0.51 and varied (13.9–16.3) and B) cord blood samples (three donors: C4, C5 and C6); average Cq was 14.92 ± 0.86 and varied (13.9–16.9). Each bar represents the average Cq-values and the error bar indicates ± SD. (PDF 33 KB) [file 13104_2014_3158_MOESM2_ESM.pdf]

Additional file 2 - Evaluation of *18S rRNA* stability

The stability of *18S rRNA* for adult and cord blood samples was analysed. Raw *Cq*–values for *18S rRNA* were relatively stable following storage of blood samples for up to six years at -80ºC. A) Adult blood samples (three donors: A1, A2 and A3); *Cq* average ± SD (and range): 14.63 ± 0.51 (13.9 – 16.3). B) Cord blood samples (three donors: C4, C5 and C6); *Cq* average ± SD (and range): 14.92 ± 0.86 (13.9 – 16.9). Average *Cq*-values and error bars (±SD) are shown.

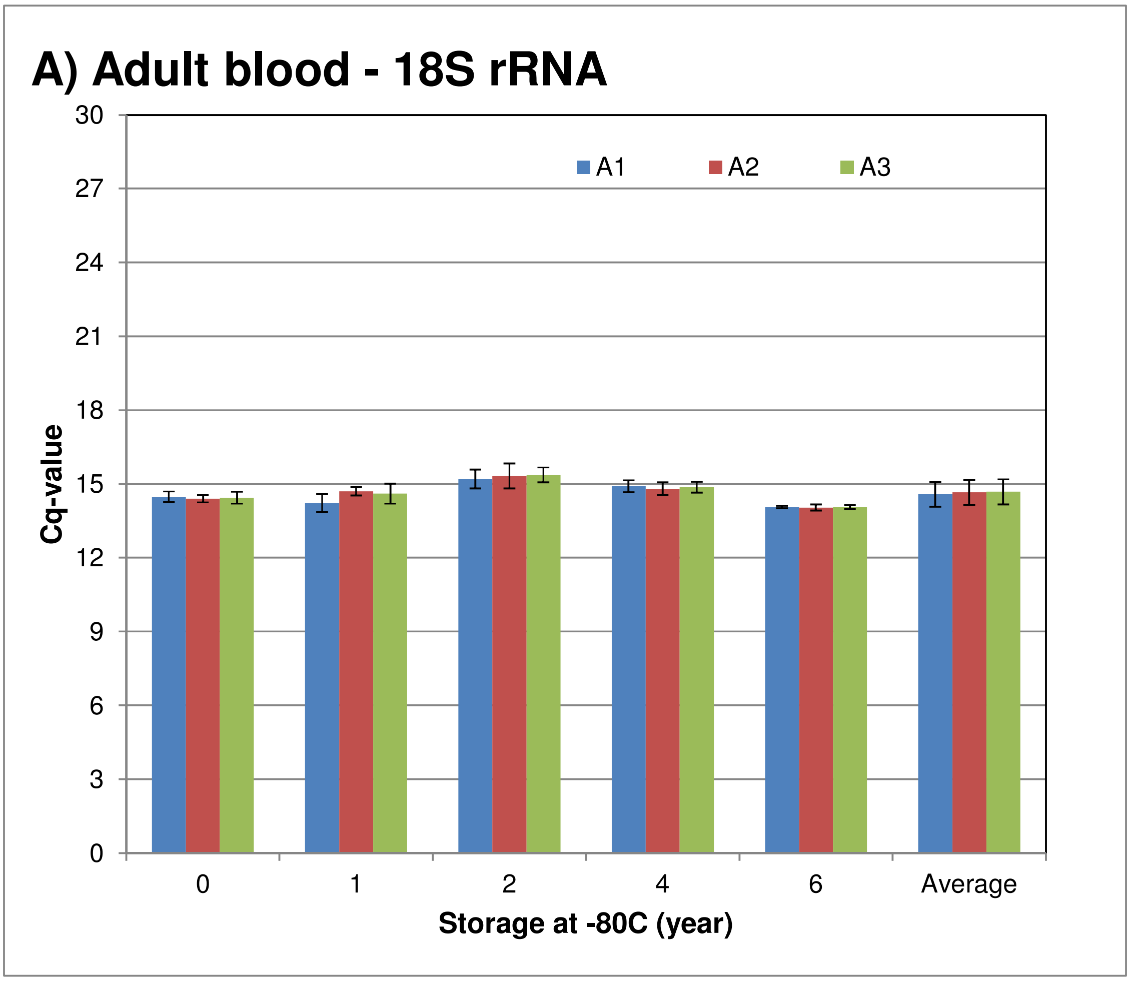

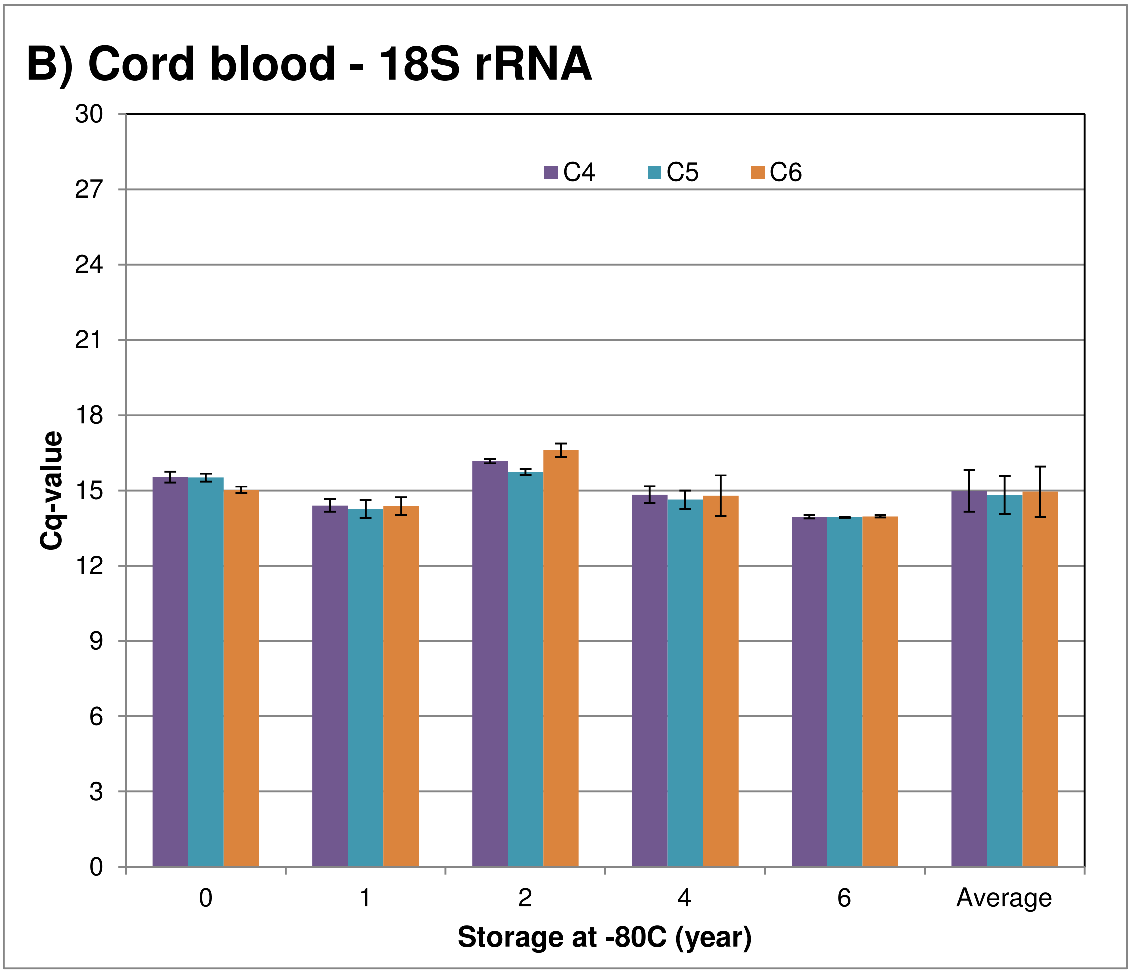

B) Cord blood - 18S rRNA
C4
C5
C6
Cq-value
30
27
24
21
18
15
12
9
6
3
0
0
1
2
4
6
Average
Storage at -80C (year)
